# Enhanced Immunogenicity of an Influenza Ectodomain Matrix-2 Protein Virus-like Particle (M2e VLP) Using Polymeric Microparticles for Vaccine Delivery

**DOI:** 10.3390/v14091920

**Published:** 2022-08-30

**Authors:** Keegan Braz Gomes, Ipshita Menon, Priyal Bagwe, Lotika Bajaj, Sang-Moo Kang, Martin J. D’Souza

**Affiliations:** 1 Vaccine Nanotechnology Laboratory, Center for Drug Delivery, Department of Pharmaceutical Sciences, College of Pharmacy, Mercer University, Atlanta, GA 30341, USA; 2 Center for Inflammation, Immunity, and Infection, Institute for Biomedical Sciences, Georgia State University, Atlanta, GA 30303, USA

**Keywords:** vaccine, virus-like particle, influenza, microparticle, live-cell imaging

## Abstract

In this study, we demonstrate how encapsulating a conserved influenza ectodomain matrix-2 protein virus-like particle (M2e5x VLP) into a pre-crosslinked bovine serum albumin (BSA) polymeric matrix enhances in vitro antigen immunogenicity and in vivo efficacy. The spray-dried M2e5x VLP-loaded BSA microparticles (MPs) showed enhanced stimulation of antigen presenting cells (APCs), as confirmed through nitrite production and increased antigen–cell interactions seen in real time using live-cell imaging. Next, to further boost the immunogenicity of M2e5x VLP microparticles, M2e5x MPs were combined with Alhydrogel^®^ and monophosphoryl lipid-A (MPL-A^®^) adjuvant microparticles. M2e5x VLP MPs and the combination VLP M2e5x VLP + Alhydrogel^®^ + MPL-A^®^ MPs elicited a significant increase in the expression of antigen-presenting molecules in dendritic cells compared to M2e5x VLP alone. Lastly, for preliminary evaluation of in vivo efficacy, the vaccine was administered in mice through the skin using an ablative laser. The M2e5x VLP + Alhydrogel^®^ + MPL-A^®^ MPs were shown to induce high levels of M2e-specific IgG antibodies. Further, a challenge with live influenza revealed heightened T-cell stimulation in immune organs of mice immunized with M2e5x VLP + Alhydrogel^®^ + MPL-A^®^ MPs. Hence, we utilized the advantages of both VLP and polymeric delivery platforms to enhance antigen immunogenicity and adaptive immunity in vivo.

## 1. Introduction

Influenza remains an ongoing public-health issue. Each year, influenza causes 3–5 million cases of severe illness and upwards of 650,000 deaths [1]. Although annual flu vaccines are readily available and can offer moderate protection, mismatched vaccine strains often result in reduced protection against rapidly mutating circulating strains [1,2]. Additionally, antigenic shift resulting in rapid reassortment of viral genomes can lead to the emergence of pandemic strains, against which most of the general population lacks pre-existing immunity [1,2]. Therefore, it is important to develop a universal influenza vaccine.

The ectodomain matrix-2 protein (M2e) of the influenza virus has been explored as a possible antigen for a universal influenza vaccine as it is highly conserved in influenza A strains. However, as M2e is only 23 amino acids, it has poor immunogenicity [3]. There have been various strategies used to improve the immunogenicity of M2e, including the use of various carriers or immunopotentiators, such as gold nanoparticles [3], hepatitis B core particles [4], liposomes [5], and flagellin [6]. An alternate strategy involves constructing recombinant M2e virus-like particles (VLPs) [7]. Previously, Kang et al. engineered an M2e construct with a tandem repeat of M2e sequences derived from human, swine, and avian influenza A viruses to offer broader protection against a range of influenza A strains. This vaccine construct, M2e5x VLP, demonstrated robust cross-protection against various HA subtypes [7].

In this study, we incorporated M2e5x VLP it into a polymeric microparticle delivery vehicle to further enhance its immunogenicity. Microparticles for vaccine delivery are advantageous as their size and shape mimic the size of bacterial pathogens, allowing for enhanced immune recognition and internalization by antigen-presenting cells (APCs) [8]. Encapsulation, adsorption, and conjugation of antigens into or onto synthetic or protein polymeric microparticles have enhanced antigen uptake and improved antigen presentation in antigen-presenting cells (APCs) [8]. In this study, we used cross-linked albumin as an antigen delivery matrix for M2e5x VLP. Albumin has previously been shown to be stable across various pH, highly soluble, and biodegradable [9].

Formulation of a polymeric particulate vaccine can involve several techniques, including spray drying, emulsification, solvent evaporation, lyophilization, and coacervation [10]. For formulating M2e5x-loaded bovine serum albumin (BSA) microparticles, spray drying was used. Spray drying is a one-step process of encapsulation, in which an antigen–polymer solution is aerosolized to create droplets that are briefly heated to evaporate the water and then cooled to produce spray-dried particulates [11]. A previous study demonstrated a spray-drying process that stabilized a whole inactivated influenza virus vaccine through modification of various parameters, such as inlet air temperature, nozzle gas flow rate, and feed rate. The process also did not negatively impact the immunogenicity of the antigen [12]. Various parameters can be optimized to produce spray-dried powders with desired particle size, residual moisture content, and product yield [12].

For an effective vaccine, immunostimulation is important for inducing a robust immune response. Dendritic cells (DCs) are professional antigen-presentation cells and are the only type of phagocytes capable of stimulating primary and memory immune responses. Upon interaction with a pathogen, DCs takes up the pathogen through receptor-mediated endocytosis through specialized regions of the cell’s plasma membrane. Particulate and soluble antigens are internalized by DCs through phagocytosis and micropinocytosis, respectively [13]. The cells then process the antigen into peptides, which are then loaded onto major histocompatibility complexes (MHCs) I and II. Next, the DCs migrate to lymphoid organs to present the antigen to T lymphocytes to drive antigen-specific immune responses [14]. In this study, we demonstrate APC–antigen interactions through live-cell imaging of dendritic cells pulsed with antigen-loaded microparticles. Further, we evaluated how M2e5x VLP polymeric microparticles can improve antigen presentation on MHC I and MHC II and their co-stimulatory molecules in DCs using flow cytometry. Another advantage of antigens delivered using a polymeric microparticle is the potential to induce cross-presentation in APCs [8].

In addition to intrinsic immunopotentiating properties, particulate delivery systems can also be loaded with adjuvants to help boost the immune response towards pathogens, such as influenza, by focusing on stimulating specific toll-like receptor (TLR) agonists [8]. Adjuvants are widely used to boost the immune response towards an antigen by targeting the immune system directly [15]. Aluminum (alum) adjuvants have been shown to induce long-lasting and protective antibodies [16]. Alum forms a depot at the site of administration when administered with a vaccine antigen and drives a Th2 response. Alum is considered one of the safest approved adjuvants for human use. Oil emulsions are another class of adjuvants that have shown to have robust immunostimulatory properties. Several of these types of adjuvants target toll-like receptors (TLRs) on the surface of immune cells, which can activate adaptive immune responses, leading to long-term protection against pathogens [17]. Monophosphoryl lipid A (MPL-A^®^), is an oil emulsion shown to improve resistance against viral infections by modulating cytokine release by specifically driving a Th1 response [18,19].

Lastly, we carried out a preliminary in vivo study to evaluate the efficacy of the vaccine microparticles delivered through the skin. We evaluated how administering vaccine microparticles through the skin using an ablative laser could potentially serve as a less-invasive, needle-free route for vaccine delivery. Laser microporation of skin to deliver vaccines has previously been shown to significantly improve antibody responses as the skin can serve as an antigen depot, allowing for slow diffusion of the vaccine into deeper layers of the skin and, therefore, extend antigen presentation [20]. Therefore, in this study, we use the novel approach of combining VLP, polymeric microparticle, spray drying, and laser ablation technologies to enhance in vitro antigen presentation and uptake and in vivo efficacy of an M2e5x VLP + Alhydrogel^®^ + MPL-A^®^ microparticulate vaccine delivered through skin.

## 2. Materials and Methods

### 2.1. Formulation of M2e5x VLP Microparticles

The antigen, M2e5x VLP, was provided by Dr. Sang-Moo Kang (Georgia State University). The construct consisted of heterologous tandem repeats of two human M2e (SLLTEVETPIRNEWGSRSN), swine M2e (SLLTEVETPTRSEWESRSS), type I avian M2e (SLLTEVETPTRNEWESRSS), and type II avian M2e (SLLTEVETLTRNGWGCRCS), as previously described [21,22]. In brief, M2e5x and an oligomer-stabilizing domain GCN4 fusion protein were linked to a transmembrane cytoplasmic domain of hemagglutinin to enhance the incorporation of tandem M2e into the VLP [22]. Sf9 cells were coinfected with recombinant baculoviruses expressing M1 and M2e5x. After propagation of proteins, cell culture supernatants were centrifuged to remove cells followed by ultracentrifugation at 30,000 rpm for 1 h to pellet M2e5x VLP. VLP was then resuspended in PBS and ultracentrifuged through a sucrose gradient for 1 h. Next, the M2e5x VLP was adsorbed onto formvar carbon-coated copper grids for 15 min. For formulating M2e5x VLP-loaded BSA polymeric microparticles, 19 mg of BSA (Fisher Scientific, Hampton, NH, USA) and 4 μL glutaraldehyde (Sigma Aldrich, St. Louis, MO, USA) were first crosslinked in 20 mL deionized water overnight under magnetic stirring. The next day, excess glutaraldehyde in the solution was neutralized by the addition of 2 mg of 1M sodium bisulfite (Sigma Aldrich). Next, 200 μL of Tween 80 was added to the mixture. Under magnetic stirring, a suspension containing 1 mg of M2e5x VLP was added dropwise to the polymer solution. The solution was then sprayed using a Büchi Mini Spray Dryer B-290 (Büchi Laboratory Equipment, Flawil, Switzerland) at 120 °C using compressed air, with a pump speed of 7% and aspiration rate of 100%. The spray-drying process involves atomization through forcing a fluid through an outlet that creates a spray that can be optimized to produce droplets of a desired size. After atomization, the moisture from the droplets is removed through drying. The spray-dried M2e5x VLP microparticles were collected in a collection vessel. Adjuvant microparticles were formulated similarly through substituting the antigen with the respective adjuvants Alhydrogel^®^ or monophosphoryl lipid A (MPL-A^®^) (InVivogen, San Diego, CA, USA) at a 5% *w/v* loading concentration.

### 2.2. Physical Characterization of Microparticles

M2e5x VLP BSA polymeric microparticles were characterized for their morphology, size, and charge. The morphology of the microparticles was visualized using scanning electron microscopy (SEM; Phenom benchtop SEM, Nanoscience Instruments, Phoenix, AZ, USA). Particle size and surface charge (zeta potential) were measured using a Malvern Zetasizer (Malvern, UK).

### 2.3. Nitric Oxide Production

To assess the induction of nitric oxide by M2e5x VLP, M2e5x VLP MPs, and adjuvant particles in immune cells, dendritic cells (DC 2.4, ATCC, Manassas, VA, USA) were plated in a 24-well plate at a density of 5 × 10^4^ cells/well. After the cells reached confluency, various treatments were introduced to the cells in 500 μL of complete DMEM (DMEM, 10% FBS, 1% antibiotics) media: Cells Only (negative control), M-Vac (5 μL marketed measles vaccine; positive control), blank (unloaded) MPs, M2e5x VLP suspension, Alhydrogel^®^ MPs, MPL-A^®^ MPs, and M2e5x VLP MPs. For M2e5x VLP, 0.5 μg total M2e5x VLP suspension was added to the wells. For M2e5x VLP MP and adjuvant MP groups, approximately 10 μg MPs (M2e5x VLP, Alhydrogel^®^, or MPL-A^®^) was added to each well. The plates were incubated for 24 h after which the Griess’ Assay was performed to assess nitrite production. Post incubation, 500 μL of cell supernatant was removed from sample wells and centrifuged at 6000 rpm for 3 min to eliminate any cellular debris. Next, 50 μL of supernatant from each sample was spun down and added to a 96-well plate. Next, 50 μL of 1% sulphanilamide was added to the wells. The plates were then covered in foil and incubated at room temperature (RT) for 5 min in the dark. Next, 50 μL of 0.1% naphthylenediamine (NED) in 5% phosphoric acid was added to each well of the plate. The plates were then covered in foil and placed on a shaker for 5 min. The plates were then read at 540 nm using a BioTek Synergy plate reader (BioTek, Winooski, VT, USA).

### 2.4. Live-Cell Imaging of Immune Cell Interactions with Vaccine Microparticles

Since antigen–cell interactions are important for uptake and presentation of antigens, a real-time live-cell imaging study was performed. The purpose of this study was to observe interactions between dendritic cells and an antigen presented in a microparticulate delivery vehicle. First, dendritic cells were seeded at 5 × 10^4^ cells/well in a 24-well plate. The next day, the cells were incubated with 16 μg of M2e5x VLP MPs in 200 μL media for 1 h. The aforementioned particle concentration was found to be optimal for visualizing dendritic cell interactions with microparticles. The cells were recorded under a brightfield lens at 60× magnification using a BioTek Lionheart Live Cell Imager (Winooski, VT, USA) for 1 h.

### 2.5. Antigen Presentation in Dendritic Cells

To assess in vitro antigen presentation, dendritic cells were plated in 24-well plates at a seeding density of 5 × 10^4^ cells/well. After reaching confluency, the cells were pulsed with various treatments: Cells Only (negative control), M2e5x VLP (0.5 μg/well), M2e5x VLP MPs (10 μg/well M2e5x VLP MPs), and M2e5x VLP + Alhydrogel^®^ + MPL-A^®^ MPs (10 μg M2e5x VLP MPs, 50 μg Alhydrogel^®^ MPs, and 5 μg MPL-A^®^ MPs). This ratio of M2e5x VLP MPs to adjuvant MPs (2:10:1) was selected as it would be the same ratio used for in vivo testing. The cells were incubated for 24 h. The cells were then gently scraped from the bottom of the wells and centrifuged for 10 min at 1200 rpm. Next, cells were resuspended in PBS and stained with either anti-mouse FITC-labeled MHC I and APC-labeled CD80 or anti-mouse PE-labeled MHC II and APC-labeled CD40. Cells were then analyzed using a flow cytometer (C6 flow cytometer, BD Accuri^TM^, Franklin Lakes, NJ, USA).

### 2.6. In Vitro Cytotoxicity

In a 96-well plate, 1 × 10^4^ dendritic cells were plated per well. After cells reached confluency, various treatments were introduced to cells: 10 μL dimethyl sulfoxide (DMSO; Sigma Aldrich, St. Louis, MO, USA), Cells Only (no treatment), and various concentrations of M2e5x VLP microparticles ranging from 31.25 to 1500 μg/mL. Each particle-containing well received 100 μL of particle suspension. For short-term cytotoxicity, the cells were treated for 24 h whereas for long-term cytotoxicity, the cells were treated for 96 h. After the incubation period, the cells were washed three times with PBS. Next, 10 μL of 0.5% *w/v* MTT reagent stock (Sigma Aldrich, St. Louis, MO, USA) was added to all wells. Thereafter, 90 μL of complete DMEM was added to each well. The plate was then incubated for 4 h to allow formation of the purple precipitate by metabolically active (live) cells. Next, media were removed and 100 μL of DMSO was added to each well. The plate was then covered in foil and placed on a shaker for 15 min to solubilize the precipitate. The plate was then read at 570 nm. The average absorbance of the cells-only group (untreated) was set as 100% cell viability. The following equation was used to calculate cell viability in other groups: average % cell viability = (absorbance of test group/absorbance of Cells Only) × 100.

### 2.7. Immunization, Blood Collection, and Challenge

For proof of concept to demonstrate how in vitro antigen presentation may translate to in vivo efficacy, 4–6-week-old Swiss Webster (CFW) mice (Charles River, Wilmington, MA, USA) were immunized as follows (*n* = 5): Naïve (negative control), Inactivated PR8 H1N1 (5 μg–positive control), and M2e5x VLP + Alhydrogel^®^ + MPL-A^®^ MPs (200 μg M2e5x VLP MPs + 1000 μg Alhydrogel^®^ MPs + 100 μg of MPL-A^®^ MPs). Two days prior to immunization, the dorsal sides of mice were shaved with a hair trimmer along with the addition of depilatory cream (Nair™, Church and Switch Co., Inc., Ewing, NJ, USA). For immunizations, the microparticles were suspended in PBS. All groups except Naïve received two applications of the Precise Laser Epidermal System (P.L.E.A.S.E.^®^) ablative laser. Laser ablation parameters consisted of a pulse duration of 125 microseconds, repetition rate of 200 Hz, 2 pulses/pore, pore diameter of 220 microns, pore density of 80 pores/cm^2^, and maximum pore depth of 2 mm. Next, the inactivated PR8 H1N1 or MP vaccine suspensions were pipetted onto newly formed micropores on the skin. Each mouse received 3 immunizations at 3-week intervals (weeks 0, 3, and 6). Blood was collected at weeks 4, 7, and 10 for assessing serum IgG. In brief, whole blood was allowed to clot at room temperature for 30 min before being centrifuged at 1500 G for 10 min. The obtained serum was stored at −80 °C until use. Six weeks after the final booster, the mice were challenged intranasally with 50 μL of an 0.5 × LD_50_ dose of A/Philippines/2/82 (H3N2) to assess T-cell responses of vaccinated mice compared to naïve mice post challenge. The mice were monitored for 14 days post challenge after which they were sacrificed.

### 2.8. M2e-Specific Antibody Response

Serum IgG was assessed using ELISA. M2e was coated on 96-well plates at a concentration of 0.2 μg/well. Inactivated PR8 H1N1 was used as a coating antigen for the inactivated PR8 group (0.2 μg/well). The plates were incubated at 4 °C overnight. The next day, plates were washed three times with PBS + 0.1% Tween 20 (PBST) and blocked with 3% BSA (100 μL/well) for 2.5 h at RT. Serum samples were thawed on ice and serially diluted 10 times in PBST starting at 1:10 dilution. The plates were then washed, and the diluted samples (50 μL/well) were added to the plates and incubated at 37 °C for 1.5 h. The plates were then washed before the addition of 50 μL/well of horseradish peroxidase (HRP)-conjugated goat anti-mouse IgG (EBiosciences, San Diego, CA, USA) at a dilution of 1:4000. The plates were then incubated at 37 °C for 1.5 h. Next, plates were washed and 50 μL of 3,3′,5,5′–Tetramethylbenzidine (TMB; BD Biosciences, Franklin Lakes, NJ, USA) was added to each well. The plates were incubated for 4 min at room temperature. The reaction was stopped by adding 50 μL/well of 0.3M sulfuric acid (Sigma Aldrich, St. Louis, MO, USA). Plate absorbance was measured at 450 nm using a plate reader.

### 2.9. T-Cell Response

After animals were challenged and sacrificed, lymph nodes (inguinal and brachial) and spleens were harvested. Single-cell suspensions of lymph nodes were prepared by passing organs through a 40 μm cell strainer. The cells were then centrifuged at 1200 rpm and resuspended in DMEM containing 70% fetal bovine serum (FBS) and 5% *v/v* DMSO and frozen at −80 °C. Single-cell suspensions of splenocytes were made by passing spleens through a 40 μm cell strainer. Lysis of red blood cells (RBCs) was carried out by adding ammonium chloride potassium (ACK) lysis buffer for 3 min. Cells were then washed and pelleted at 1200 rpm. Next, the cells were resuspended in freezing media and stored at −80 °C until analysis. For determining expression of CD4 and CD8 in lymphocytes and splenocytes, the cell suspensions were first thawed on ice and resuspended in PBS. Cells were then stimulated with 5 μg/mL IL-2 overnight and then labeled with anti-mouse FITC-labeled CD4 and APC-labeled CD8 antibodies. After 1 h incubation on ice, the cells were washed 3x and analyzed using flow cytometry.

### 2.10. Statistical Analysis

Data analyses were performed on GraphPad Prism Version 9.2. and expressed as mean values with a standard deviation (SD). For multiple comparisons, one-way analysis of variance (ANOVA) was performed with Tukey’s test for post hoc analysis. A *p*-value < 0.05 was considered to be statistically significant.

## 3. Results

### 3.1. Physical Characterization of Microparticles

**Figure 1:**
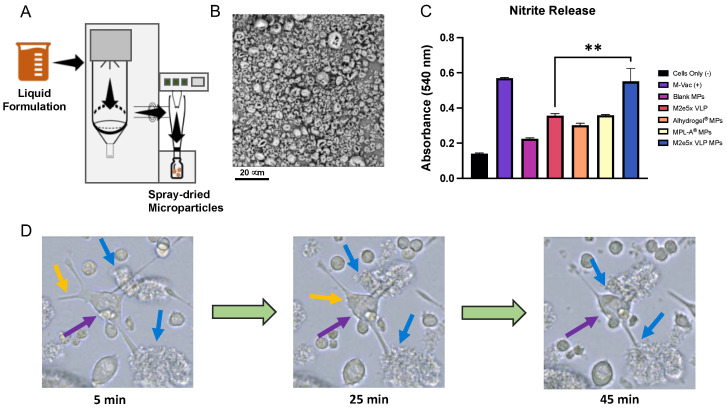
Formulation and in vitro immunogenicity of M2e5x VLP MPs. (**A**) Schematic of spray-drying process used to produce M2e5x VLP and adjuvant microparticles. (**B**) SEM image of M2e5x VLP MPs with scale bar of 20 μm. (**C**) Nitrite release indicates that M2e5x VLP MPs produce significantly higher amounts of nitrite compared to M2e5x VLP alone (** *p* < 0.01). (**D**) Brightfield images (60× magnification) to visualize antigen uptake in dendritic cells during live-cell imaging. At 5 min post treatment, cells are in contact with clusters of M2e5x VLP MPs. The purple arrow indicates the cell body of the dendritic cells, blue arrows indicate two M2e5x VLP MP clusters, and the yellow arrow indicates a highlighted dendrite. At 25 min, the highlighted dendrite at 5 min is internalized and the other dendrites begin to pull the MP clusters closer to the cell body. At 45 min, the cell body is in direct contact with an MP cluster to facilitate potential antigen uptake. A video time-lapse of this interaction is included in Supplementary Materials (Supplementary Video S1).

The yield of M2e VLP-loaded BSA microparticles after spray drying was 92.4%. A Büchi Mini Spray Dryer B-290 setup is shown in Figure 1A. The particles were approximately 4.84 ± 0.353 μm in size. The zeta potential of the M2e5x VLP MPs was found to be −19.4 ± 1.44 mV. The particles had a non-spherical “doughnut shaped” morphology, as seen in Figure 1B.

### 3.2. Nitric Oxide Production

The production of nitric oxide by immune cells is one indicator of innate immunity, which can be a precursor for stimulating an immune response. For the Griess’ assay, the nitrite ions react in the Griess diazotization reaction to form a diazonium salt, which then reacts with N-(1-naphthyl)ethylenediamine in an azo coupling reaction, forming a pink-red azo dyenitric oxide that is oxidized to nitrite, which can be measured at an absorbance of 540 nm. Nitrite results are shown in Figure 1C. All MP groups produced nitrite. However, the M2e5x VLP MPs are the only MP group that produced significantly higher levels of nitrite compared to the M2e5x VLP suspension (** *p* < 0.01).

### 3.3. Live-Cell Imaging of Immune Cell Interactions with Vaccine Microparticles

Live-cell imaging at 60× magnification of antigen uptake by dendritic cells was visualized at various time points: 4, 25, and 45 min, as shown in Figure 1D. Purple arrows identify the dendritic cell body, blue arrows depict two distinct microparticle clusters, and the yellow arrow represents a dendrite that is internalized back into the cell while the two remaining dendrites pull the antigen microparticle clusters towards the cell body. As time progresses, the cell’s dendrites pull the particle clusters closer to the cell body for potential uptake and processing. Therefore, cells pulsed with M2e5x VLP microparticles showed a strong affinity for the particles, as visualized by the capture of the particle clusters and the rearrangement of dendritic projections to better interact with the particles.

### 3.4. Antigen Presentation in Dendritic Cells

**Figure 2:**
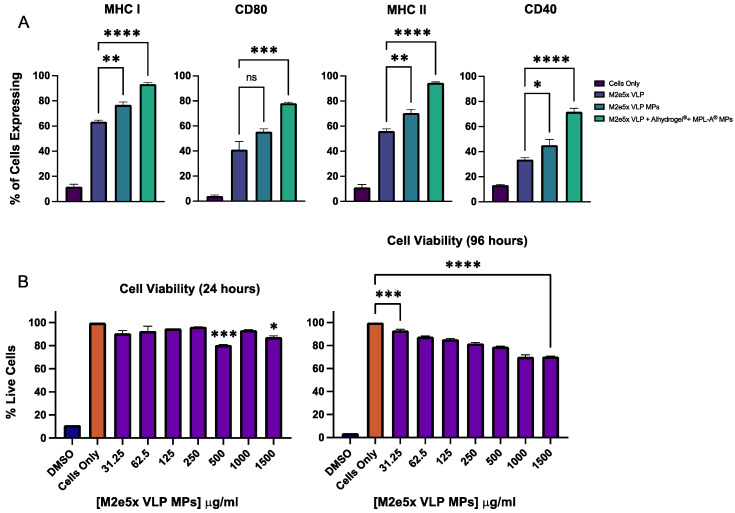
In vitro antigen presentation and cytotoxicity of MP vaccine. (**A**) There is a significantly higher expression of MHC I in M2e5x VLP MPs (** *p* < 0.01) and M2e5x VLP + Alhydrogel^®^ + MPL-A^®^ MPs (**** *p* < 0.0001) compared to M2e5x VLP alone. For CD80 expression, M2e5x VLP + Alhydrogel^®^ + MPL-A^®^ MPs (*** *p* < 0.001) shows significantly higher expression compared to M2e5x VLP. For MHC II, M2e5x VLP MPs (** *p* < 0.01) and M2e5x VLP + Alhydrogel^®^ + MPL-A^®^ MPs (**** *p* < 0.0001) showed significantly higher expression compared to M25xe VLP alone. Lastly for CD40 expression, M2e5x VLP MPs (* *p* < 0.05) and M2e5x VLP + Alhydrogel^®^ + MPL-A^®^ MPs (**** *p* < 0.0001) elicited significantly higher CD40 expression compared to M2e VLP alone. (**B**) At 24 h, there is a statistical difference in cell viability between 500 μg/mL (*** *p* < 0.001) and 1500 μg/mL (* *p* < 0.05) particle concentrations compared to cells only control. At 96 h, there is a statistical difference between all concentrations compared to Cells Only. All cell viabilities for all M2e5x VLP MP concentrations after 24 h and 96 h were at least 78% and 68%, respectively.

For antigen presentation, the percent of DCs expressing MHC I, CD80, MHC II, or CD40 is shown in Figure 2A. There is a significantly higher expression of MHC I in M2e5x VLP MPs (** *p* < 0.01) and M2e5x VLP + Alhydrogel^®^ + MPL-A^®^ MPs (**** *p* < 0.0001) compared to M2e5x VLP. CD80 expression in DC’s is significantly higher in M2e5x VLP + Alhydrogel^®^ + MPL-A^®^ MPs (*** *p* < 0.001) compared to M2e5x VLP. Expression of MHC II in M2e5x VLP MPs (** *p* < 0.01) and M2e5x VLP + Alhydrogel^®^ + MPL-A^®^ MPs (**** *p* < 0.001) is also significantly higher than M2e5x VLP. Lastly, for CD40 expression, M2e5x VLP MPs (* *p* < 0.05) and M2e5x VLP + Alhydrogel^®^ + MPL-A^®^ MPs (**** *p* < 0.001) are both statistically higher than M2e5x VLP. In conclusion, M2e5x VLP encapsulated in BSA microparticles (M2e5x VLP MPs) resulted in a significant increase in antigen presentation in MHC I, MHC II, and CD40 compared to M2e5x VLP alone. Additionally, the incorporation of adjuvants further improves the antigen presentation of the M2e5x VLP MPs as M2e VLP + Alhydrogel^®^ + MPL-A^®^ MPs demonstrated significantly higher expressions of MHC I, CD80, MHC II, and CD40 compared to M2e5x VLP alone.

### 3.5. In Vitro Cytotoxicity

Cell toxicity of M2e5x VLP MPs is shown in Figure 2B. For M2e5x VLP MPs, cell viability (% of negative control) was not concentration dependent after treatment for 24 h, with all MP concentrations demonstrating cell viabilities of at least 78%. Only 500 μg/mL (*** *p* < 0.001) and 1500 μg/mL (* *p* < 0.05) were statistically different from Cells Only. The 1000 μg/mL concentration did not result in a significant reduction in cell viability compared to Cells Only. After 96 h exposure to M2e5x MPs, cell viability was concentration dependent, with higher MP concentrations demonstrating decreased cell viability. Cell viability for all M2e5x MP concentrations at 96 h was statistically lower than Cells Only. However, cell viabilities for all groups remained above 68%.

### 3.6. M2e-Specific Antibody Response

**Figure 3:**
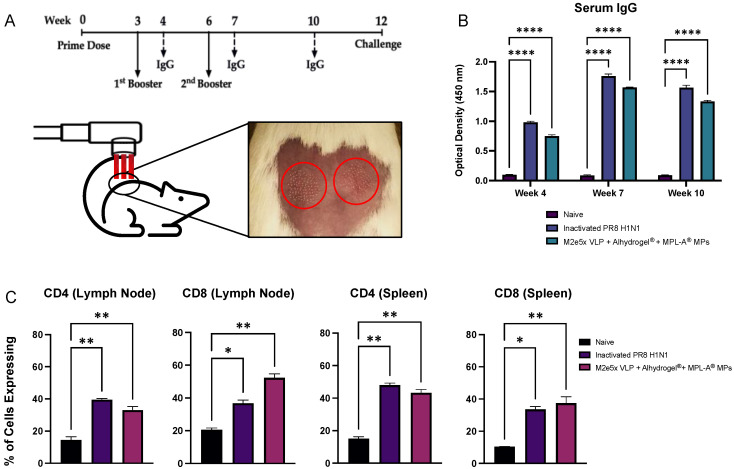
In vivo evaluation of adjuvanted M2e5x VLP MP vaccine. (**A**) Study design and laser ablation device used for immunizations in mice. The red circles highlight the micropores created by the P.L.E.A.S.E.^®^ ablative laser. (**B**) Serum IgG levels for weeks 4, 7, and 10 comparing Inactivated PR8 H1N1 and M2e5x VLP + Alhydrogel^®^ + MPL-A^®^ MPs to Naïve showed that mice that received monovalent inactivated PR8 (**** *p* < 0.0001) and the experimental MP vaccine (**** *p* < 0.0001) produced significantly higher IgG levels compared to naïve mice. The experimental vaccine, containing the antigen (M2e5x VLP) and adjuvant (Alhydrogel^®^ + MPL-A^®^) MPs was shown to induce high levels of M2e-specific antibodies in vivo. (**C**) CD4^+^ and CD8^+^ T cells in lymph node and splenocyte populations in Inactivated PR8 H1N1 and M2e5x VLP + Alhydrogel^®^ + MPL-A^®^ BSA MPs were significantly higher compared to Naive. *p*-values: * *p* < 0.05, ** *p* < 0.01， and **** *p* < 0.0001.

The dosing regimen, blood collection, and challenge schedule are shown in Figure 3A. A schematic of the P.L.E.A.S.E.^®^ ablative laser and microporated skin after laser treatment is also shown in Figure 3A. Serum IgG results are shown in Figure 3B. Mice immunized with M2e5x VLP + Alhydrogel^®^ + MPL-A^®^ MPs produced significant levels of M2e-specific antibodies compared to Naive. Inactivated PR8 H1N1-immunized mice (positive control) produced significant levels of PR8 H1N1-specific IgG. Hence, both Inactivated PR8 H1N1 and M2e5x VLP + Alhydrogel^®^ + MPL-A^®^ MPs demonstrated significantly higher IgG compared to the naïve group (**** *p* < 0.0001) at week 4 (post-first boost) and week 7 (post-second boost), remaining elevated through week 10.

### 3.7. T Cell Response

The challenge was not lethal for all mice, with no significant weight loss observed. CD4 and CD8 T-cell responses in lymph nodes (inguinal and brachial) and splenocytes are shown in Figure 3C. Inactivated PR8 H1N1 and M2e5x VLP + Alhydrogel^®^ + MPL-A^®^ BSA MPs showed heightened expression of CD4 and CD8 in T cells in spleen and lymph nodes compared to Naïve, which resembled the T-cell response to influenza infection in non-immunized mice. In lymph nodes, Inactivated PR8 H1N1 (** *p* < 0.01) and M2e5x VLP + Alhydrogel^®^ + MPL-A^®^ BSA MPs (** *p* < 0.01) showed significantly higher percentages of CD4^+^ cells compared to Naïve. For CD8 expression, similarly, both Inactivated PR8 H1N1 (* *p* < 0.05) and M2e5x VLP + Alhydrogel^®^ + MPL-A^®^ BSA MPs (** *p* < 0.01) showed significantly higher percentages of CD8^+^ T cells in lymph nodes compared to Naive. In splenocyte populations, Inactivated PR8 H1N1 (** *p* < 0.01) and M2e5x VLP + Alhydrogel^®^ + MPL-A^®^ BSA MPs (** *p* < 0.01) also exhibited significantly higher percentages of CD4^+^ cells compared to Naïve. Lastly, for CD8^+^ T cells in the spleen, Inactivated PR8 H1N1 (* *p* < 0.05) and M2e5x VLP + Alhydrogel^®^ + MPL-A^®^ BSA MPs (** *p* < 0.01) showed significantly higher percentages of cell expressing CD8 compared to Naïve.

## 4. Discussion

M2e was selected as the vaccine antigen as it is highly conserved in influenza A strains. The production of M2e-specific antibodies, although non-neutralizing, can confer cross-protection against various influenza strains by M2e antibody-dependent cell cytotoxicity (ADCC) [23]. Resultantly, a successful vaccine priming with M2e could lead to a potential universal influenza A vaccine. However, as M2e is not highly immunogenic, its immunogenicity was increased by combining M2e in tandem repeats to produce M2e5x VLP. The M2e in a VLP form is more immunogenic as it better resembles the influenza virion while containing a high density of M2e epitopes [7]. Next, the incorporation of the antigen into a BSA microparticulate matrix conferred several advantages. First, the negative zeta potential of the microparticles indicates uniform size distribution and vaccine stability. Previous studies have shown that high negative or positive zeta potential indicates a uniform size distribution [24]. Furthermore, highly charged particles do not agglomerate as easily, resulting in greater vaccine stability [24]. Next, the size of the M2e5x VLP microparticles (approximately 5 μm) is advantageous for particle uptake and antigen presentation. Microparticles between 1 and 5 μm in size have previously been shown to be efficiently phagocytosed by specialized phagocytes, thereby inducing a robust immune response [25]. The encapsulation of M2e5x VLP into BSA polymeric microparticles also enhanced production of nitric oxide in dendritic cells compared to M2e5x VLP alone. Nitric oxide can be used as an indicator of innate immunity. Production of nitric oxide is often central to clearance of pathogenic infections prior to the activation of adaptive immunity [26]. Therefore, the significant increase in nitrite through encapsulation of M2e VLP in a polymeric matrix is indicative of a potential enhancement in immune system activation.

Dendritic cell interactions with M2e5x VLP MPs were further assessed through real-time live-cell imaging as seen through dendrite rearrangements and dendritic pull of microparticle clusters. The live-cell imaging study confirmed that micron-sized particles can visibly enhance activity, interactions, and uptake by the dendritic cell body. This affinity is likely due to the size and irregular shape of the microparticles, as this high dendrite activity and reorganization was not seen in cells pulsed with M2e5x VLP alone (not shown). Additionally, the microparticle polymer itself, BSA, has been shown to have self-adjuvanting properties [24,27], which can possibly explain enhanced affinity towards dendritic cells. To further confirm whether these cell–antigen interactions correlate to enhanced phagocytosis and presentation, antigen presentation molecules were analyzed. The increased expression of MHC I, MHC II, and CD40 molecules in M2e5x VLP MP-pulsed DCs compared to M2e5x VLP alone suggests that an increase in antigen presentation occurs when the antigen is presented in a polymeric microparticulate delivery vehicle. Furthermore, when this delivery system is combined with adjuvant microparticles, there is a significant increase in MHC I, CD80, MHC II, and CD40 expression. The improved presentation of MHC I in dendritic cells in response to both M2e VLP MPs and M2e VLP + adjuvant MPs demonstrates a strong case for cross-presentation.

Past studies have shown that the use of erodible microparticles containing antigen can act as an antigen depot upon contact with APCs, resulting in both intracellular and extracellular release of the antigen that prolongs antigen availability [28]. The delivery of an antigen to the cytoplasm versus endosomal compartments of the antigen-presenting cell also directs a specific pattern for MHC presentation. Endocytosed proteins are degraded in the endo-lysosomal compartments and loaded onto MHC II and presented at the cell surface to other APCs, including B cells, macrophages, and DCs. In contrast, cytosolic proteins cleaved by the proteasome are transported to the endoplasmic reticulum (ER) and presented onto MHC I molecules, which is accessible by cytolytic CD8^+^ T cells. [29]. Therefore, significant increases in MHC I, CD80, MHC II, and CD40 expression observed in this study suggest that the particle delivery system with adjuvants elicits overall improved antigen presentation but also specifically enhances cross-presentation in dendritic cells. These results are supported by previous findings from our group, which demonstrated the advantages of antigen and adjuvant encapsulation into polymeric microparticles to improve antigen presentation and overall in vivo efficacy for various vaccine antigens [21,30,31,32]. Additionally, the M2e VPL MPs were well tolerated in cells at concentrations up to 1500 μg/mL for up to 96 h (4 days).

As a proof of concept to explore if enhanced in vitro antigen presentation may translate to in vivo efficacy, mice were immunized through the skin with both an inactivated PR8 H1N1 (positive control) and the proposed MP vaccine consisting of M2e5x VLP + Alhydrogel^®^ + MPL-A^®^ MPs. ELISA results showed that immunizations with M2e5x VLP + Alhydrogel^®^ + MPL-A^®^ MPs were able to induce significant levels of M2e-specific antibodies. A similar trend was seen in CD4^+^ and CD8^+^ T-cell expression in lymphocyte and splenocyte populations of immunized mice post challenge. These preliminary results indicate that the vaccine microparticles administered through laser-microporated skin have the potential to elicit a strong immune response in vivo. This robust immune response is likely linked to large populations of Langerhans cells and dermal dendritic cells in the skin that can take up, process, and present the antigen to induce an immune response [33]. Additionally, skin immunization has previously been shown to be more potent and dose sparing compared to other immunization routes [34]. Therefore, exploring vaccination through the skin using antigen- and adjuvant-loaded polymeric microparticles will be beneficial to future vaccine development. We recently demonstrated a dual-delivery vaccination platform that uses fast-dissolving microneedles to deliver antigen-loaded polymeric particulates into various skin types [35].

Future studies will address limitations of this current study pertaining to antibody and cellular responses in mice. Follow-up studies will address the role of antigen- and adjuvant-loaded polymeric microparticles on Th1/Th2-skewed, cytokine, and M2e-specific antibody/T-cell responses in M2e VLP MP groups compared to M2e VLP alone. Additionally, cross-subtype protection and potential immune responses towards the protein microparticles will also be assessed.

## 5. Conclusions

The M2e5x VLP formulated into polymeric microparticles and combined with adjuvants was shown to be a potential influenza vaccine candidate. The single-step spray-dried M2e5x VLP MP formulation was characterized for its properties, including shape, size, and charge. Furthermore, the M2e5x VLP MPs elicited significant nitrite production in dendritic cells. Furthermore, live-cell imaging demonstrated dendritic cells exhibiting a high affinity towards M2e VLP MPs. The final vaccine formulation consisting of M2e5x VLP + Alhydrogel^®^ + MPL-A^®^ MPs demonstrated enhanced in vitro antigen presentation and an increase in IgG (M2e-specific) and CD4^+^/CD8^+^ T cells in immunized mice. Hence, the incorporation of M2e5x VLP into an MP matrix with adjuvants and delivered non-invasively using laser ablation on the skin shows promise for the future development of a universal flu vaccine, capable of eliciting both innate and adaptive immunity against influenza.

## Data Availability

The data presented in this study are available upon request from the corresponding author.

## Supplementary Materials

The following supporting information can be downloaded at: https://www.mdpi.com/article/10.3390/v14091920/s1, Video S1: Live-cell imaging of particle-dendritic cell interactions. Video timelapse of a dendritic cell interacting with M2e5x VLP MP clusters. A third dendrite is internalized to maximize interactions of two remaining dendrites with microparticles. As time progresses, microparticle clusters are pulled closer to the cell body by the dendrites.
